# Genetic associations and parent-of-origin effects of *PVRL1* in non-syndromic cleft lip with or without cleft palate across multiple ethnic populations

**DOI:** 10.4178/epih.e2024069

**Published:** 2024-08-09

**Authors:** Ji Wan Park, Geon Kang, Seung-Hak Baek, Young Ho Kim

**Affiliations:** 1 Department of Medical Genetics, Hallym University College of Medicine, Chuncheon, Korea; 2 Department of Orthodontics, Seoul National University School of Dentistry, Seoul, Korea; 3 Department of Orthodontics, Institute of Oral Health Science, Ajou University School of Medicine, Suwon, Korea

**Keywords:** Case-parent trio design, Nonsyndromic cleft lip with or without cleft palate, Parent-of-origin effect, Human poliovirus receptor related 1, Transmission disequilibrium test

## Abstract

**OBJECTIVES:**

This study investigated the associations of *PVRL1* gene variants with non-syndromic cleft lip with or without cleft palate (NSCL/P) by evaluating transmission distortion and parent-of-origin (POO) effects in multiple ethnic populations.

**METHODS:**

We conducted allelic and genotypic transmission disequilibrium tests (TDT) on 10 single-nucleotide variants (SNVs) in *PVRL1* using data from 142 Korean families with an affected child. POO effects were analyzed using the POO likelihood ratio test, comparing transmission rates of maternally and paternally inherited alleles. To assess generalizability and ethnic heterogeneity, we compared results from Korean families with data from the Center for Craniofacial and Dental Genetics, which included 2,226 individuals from 497 European and 245 Asian trios.

**RESULTS:**

TDT analysis identified significant over-transmission of the rs7940667 (G361V) C allele in Korean families (p=0.007), a finding replicated in both Asian (p=6.5×10^-7^) and European families (p=1.6×10^-10^). Eight SNVs showed strong TDT evidence in larger Asian and European datasets after multiple comparison corrections (p<0.0073). Of these, 4 SNVs (rs7940667, rs7103685, rs7129848, and rs4409845) showed particularly robust association (p<5×10^-8^). POO analysis revealed significant maternal over-transmission of the rs10790330-A allele in Korean families (p=0.044). This finding was replicated in European families (p=9.0×10^-4^). Additionally, 3 other SNVs, rs7129848 (p=0.001) and the linked SNVs rs3935406 and rs10892434 (p=0.025), exhibited maternal over-transmission in the validation datasets.

**CONCLUSIONS:**

Our findings provide robust evidence supporting the associations of *PVRL1* variants with NSCL/P susceptibility. Further research is necessary to explore the potential clinical applications of these findings.

## GRAPHICAL ABSTRACT

[Fig f3-epih-46-e2024069]

## Key Message

• In a large-scale, multi-ethnic study of 2,586 individuals from Korean, Asian, and Caucasian families, we identified a significant association between *PVRL1* gene variants and the risk of non-syndromic cleft lip with or without cleft palate (NSCL/P) using transmission disequilibrium test and parent-of-origin effect analysis.

• Specifically, significant over-transmission of the rs7940667 (G361V)-C allele to affected offspring and maternal over-transmission of the rs10790330-A allele suggest potential epigenetic effects on NSCL/P risk.

• This is the first study to investigate these associations and parent-of-origin effects in a Korean population, validating the findings through a multi-ethnic approach and reinforcing their importance for disease risk prediction and genetic counseling.

## INTRODUCTION

Oral clefts, which occur due to the incomplete fusion of facial processes and/or palatal shelves during embryonic development, are categorized into 2 etiologically distinct subtypes: clefts of the lip with or without cleft palate (CL/P) and cleft palate only (CP) [1]. The most common isolated form of oral clefts is non-syndromic cleft lip with or without cleft palate (NSCL/P), affecting about 135,000 infants worldwide each year. This condition can present in various forms, including cleft alveoli, cleft lip only, and cleft lip with palate. It is significantly more prevalent among American Indians and Asians, with rates often reaching 1 in every 500 births [2]. In Korea, the overall birth prevalence of oral clefts is reported to be 1.96 per 1,000 babies, with 76.5% of these being nonsyndromic. Of these cases, CL/P accounts for approximately 53%, resulting an NSCL/P prevalence of about 0.8 per 1,000 live births [3].

NSCL/P is a craniofacial anomaly arising from a complex interplay of genetic and environmental factors. Numerous genetic loci associated with NSCL/P, including *MSX1*, *TGFA*, *IRF6*, and *PAX7*, have been identified through large-scale population-based studies [4,5]. However, the inconsistent replication of results across studies indicates that different sets of genes may be involved, or that the effects of specific genetic variants may vary among various ethnic groups.

An initial investigation of families from Margarita Island revealed a rare homozygous loss-of-function mutation (W185X) in the poliovirus receptor-related 1 (*PVRL1*) gene, also known as nectin cell adhesion molecule 1 (*NECTIN1*), located at 11q23.3, as the underlying cause of autosomal recessive CL/P-ectodermal dysplasia syndrome (CLPED1, also known as orofacial cleft 7 or Zlotogora-Ogur syndrome). It disrupts the function of the pvrl1 protein, which is crucial for cell adhesion [6]. Further mutation analyses have shown that heterozygosity for this mutation occurred more frequently in NSCL/P patients from northern Venezuela and have identified additional functional mutations in *PVRL1*, including G361V and V395M, in populations such as Caucasians, Venezuelans, and Thais [7-10].

Despite the identification of over 40 risk loci for NSCL/P through genome-wide association studies (GWASs), variants in the *PVRL1* gene have not been recognized as a significant genetic factor for NSCL/P [4,11-15]. Targeted case-control studies exploring the link between *PVRL1* gene variants and CL/P have produced inconsistent findings across various ethnic groups. For example, research involving Japanese, Taiwanese, and Han Chinese populations has not consistently demonstrated this association, suggesting genetic heterogeneity and a potentially marginal role of *PVRL1* in NSCL/P development [16-20]. While recent studies have reported parent-of-origin (POO) effects for candidate genes involved in NSCL/P, such as *TGFA* and *MSX1* [21-23], not such effects have been reported for *PVRL1* .

To the best of our knowledge, this study is the first to investigate potential POO effects of *PVRL1* gene variants in relation to NSCL/P and to evaluate the genetic association of this gene in a Korean population. The investigation was conducted in 2 phases. Initially, we examined the associations and POO effects of *PVRL1* gene variants in Korean case-parent trios. Subsequently, we replicated these findings in a multi-ethnic dataset that included European and Asian families.

## MATERIALS AND METHODS

### Study subjects

**Figure 1.**
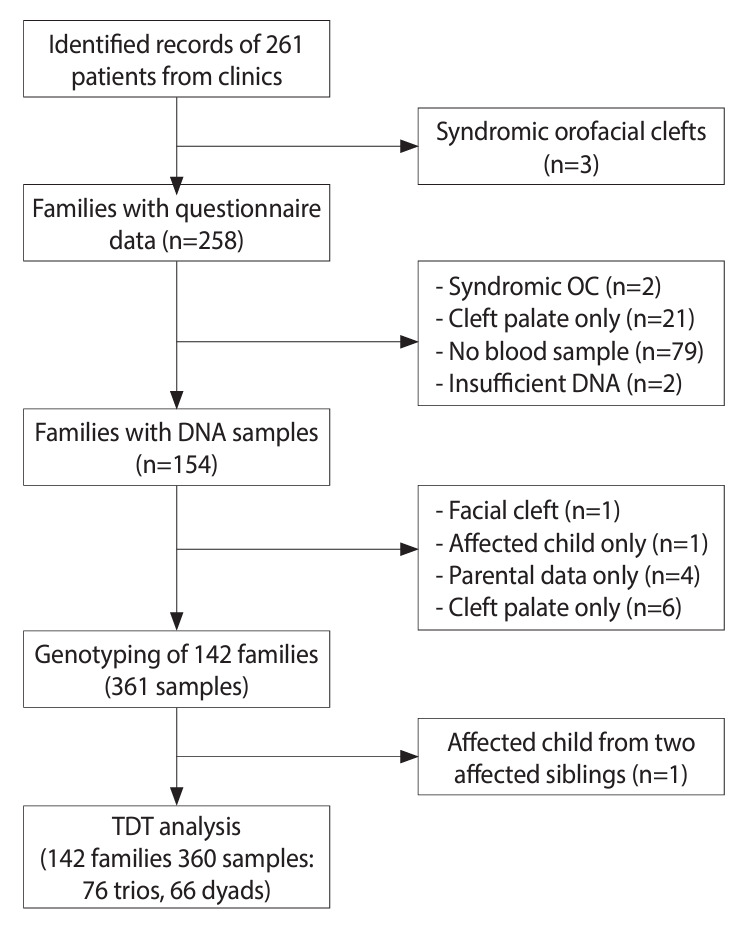
Flowchart depicting the study design and sample selection process for the Korean family trios of non-syndromic cleft lip with or without cleft palate. n, number of families; OC, oral cleft; TDT, transmission disequilibrium test.

This study involved patients from 2 centers in Seoul, Korea: Seoul National University Dental Hospital and Samsung Medical Center. Initially, the clinical records of 261 patients were identified and reviewed by dentists to confirm eligibility, focusing specifically on the diagnosis of non-syndromic oral clefts. Trained dentists then conducted structured interviews with the parents to gather clinical, demographic, and environmental data. Written informed consent was obtained from each parent before the interview. A total of 258 Korean families participated in the study, all providing complete questionnaire data (Figure 1).

To validate our findings using an independent dataset and explore ethnic differences, we accessed data from the Center for Craniofacial and Dental Genetics (CCDG): Genetics of Orofacial Clefts and Related Phenotypes (dbGaP Study Accession: phs000774. v2.p1 and phs000774.v2.p1.c1) via the Database of Genotypes and Phenotypes (dbGaP) at the National Center for Biotechnology Information (NCBI, USA). To minimize misclassification due to variations in research protocols and to mitigate the impact of potential batch effects, we concentrated our analysis on family trios from the largest single dataset available, the Pittsburgh Cohort. We included only trios with a confirmed diagnosis of NSCL/P and complete data for all 3 members (mother, father, and affected child). Trios were excluded if any family member had incomplete demographic or clinical data, high genotype missingness (> 5%), or Mendelian inheritance errors, which occur when the observed genotypes in the affected children cannot be derived from the genotypes of their parents. This led to a final sample size of 2,226 individuals, consisting of 497 European and 245 Asian family trios, including 114 Chinese trios but no Korean trios [13].

### Genotyping

We genotyped 361 individuals from 142 Korean families affected by NSCL/P, excluding those with syndromic oral clefts, CP, or insufficient DNA samples (Figure 1). These families comprised 9 cases of cleft lip, 26 cases of cleft lip and alveolus, and 107 cases of cleft lip and palate. For the genetic association study, 10 mL of peripheral venous blood was collected from each parent and child. Genomic DNA was isolated from the whole blood using a commercial kit from Qiagen Inc. (Valencia, USA). Single-nucleotide variants (SNVs) within a 4 kb region, spanning 2 kb upstream and 2 kb downstream of the *PVRL1* gene, were identified using the NCBI dbSNP database (http://www.ncbi.nlm.nih.gov/SNP/), informed by previous literature. From this initial set, candidate markers were selected from the Japanese population in dbSNP based on the following criteria: a minor allele frequency (MAF) greater than 5% and a pairwise linkage disequilibrium (LD) threshold of *r*^2^ < 0.8, as determined by the “TAG SNP selection (TagSNP)” web tool (https://snpinfo.niehs.nih.gov/snpinfo/snptag.html) [25]. The selection criteria included high “design scores” and heterozygosity above 0.1 in Asian populations (https://www.ncbi.nlm.nih.gov/snp/). Genotyping was performed by SNP Genetics, Inc. (Seoul, Korea) using Illumina’s Vera Code Technology [24].

To ensure data quality, we initially filtered the genotype data, retaining only individuals with high sample (≥ 92.6%) and genotype (≥ 99.8%) call rates. We calculated the MAF and performed the χ^2^-test for Hardy-Weinberg equilibrium (HWE) at each SNV using only parental data to characterize each population. LD was evaluated for SNV pairs within 500 kb using Haploview, employing both *D*’ and *r*^2^ measures (https://www.broadinstitute.org/haploview/haploview) [26]. SNVs with a MAF greater than 1% and an HWE p-value greater than 0.001 were included in subsequent analyses. Mendelian errors were identified using the “trio.check” function of the “trio” package v. 3.42.0. (https://bioconductor.org/packages/release/bioc/html/trio.html) [27], and families with such errors were removed from subsequent analyses to maintain data integrity. We analyzed 10 SNVs in 360 Korean samples, excluding 1 affected child from a family with 2 affected siblings.

Genotype data for Asian and European families were obtained through dbGap authorized access to the University of Pittsburgh’s CCDG dataset (dbGap Study Accession: phs000774.v2.p1). Genotyping was carried out using Infinium HumanCore BeadChips at the Center for Inherited Disease Research. Subsequent genotype cleaning was performed by the University of Washington and the NCBI. Additional information about the CCDG data is available on the dbGap website (https://www.ncbi.nlm.nih.gov/gap/?term=phs000774.v2.p1) [11].

### Statistical analysis

#### Descriptive analysis

This study included 884 families, comprising a total of 2,586 individuals divided into 3 ethnic groups: 360 Koreans, 735 Asians, and 1,491 Europeans (Table 1). The analysis explored age and sex differences among these groups, focusing on both affected children and their parents, and further breaking down the data by sex. Sex differences were evaluated using the χ^2^-test, while age distributions across the groups were compared using Welch analysis of variance to identify significant differences in group means. This was followed by the Games-Howell post-hoc test for pairwise comparisons to identify specific differences between groups [28].

#### Transmission disequilibrium test

Transmission distortion at individual SNVs was examined in 142 families, comprising 76 case-parent trios and 66 dyads (Figure 1). Allelic transmission disequilibrium testing (TDT) was performed by comparing the number of families with the minor allele transmitted to offspring versus those with the minor allele not transmitted. Counts of families with the minor allele transmitted or not transmitted to offspring were derived using Plink version 1.9. (https://www.cog-genomics.org/plink/1.9/) [29]. Genotypic TDT was assessed using conditional logistic regression models under 3 genetic models: additive, dominant, and recessive. For each SNV, the genotypic odds ratio (OR), 95% confidence interval (CI), and p-value were calculated using matched sets consisting of the case and 3 “pseudosib” controls derived from the parental mating type. Both allelic and genotypic TDT analyses were performed using the “trio” package [27]. In cases where initial TDT showed under-transmission of the minor allele, both allelic and genotypic TDT were conducted using the major allele, which showed overtransmission, to further explore potential associations. To adjust for multiple testing of SNVs in LD, we applied a significance threshold of p<0.0073, determined using the single nucleotide polymorphism spectral decomposition (SNPSpD) method [30].

#### Analysis of parent-of-origin effects

To assess POO effects, we compared the transmission rates of maternally and paternally inherited minor alleles using the POO likelihood ratio test developed by Weinberg [31]. This analysis was performed using the “colPOlrt” function implemented in the “trio” package [27]. ORs were calculated based on the number of families in which the minor allele was either transmitted or not transmitted to the offspring, using data derived from Plink version 1.9. A maternal POO effect was deemed significant if the minor allele was transmitted from the mother to the child significantly more often than from the father.

#### Replication analysis

To validate the genetic association identified in Korean families and explore potential ethnic heterogeneity, we replicated our analyses using independent datasets from Asian and European families. These datasets included 2,226 individuals from 497 European and 245 Asian family trios, which encompassed 114 Chinese trios but no Korean trios. We applied consistent quality control procedures and statistical analysis methods, as previously described, to both the initial and replication datasets. This approach ensured a reliable comparison of TDT and POO analyses.

### Ethics statement

This study adhered to the ethical guidelines outlined in the Declaration of Helsinki. Written informed consent was obtained from all participants prior to their participation in the study. The study protocol was approved by the Institutional Review Boards of Seoul National University Dental Hospital (SNUDH IRB CRI-G07002), Samsung Biomedical Center (SMC IRB #2007-08-086), and Hallym University (HIRB-2021-040).

## RESULTS

### Baseline characteristics of study subjects

The flowchart outlines the stages of participant recruitment, inclusion criteria, and reasons for exclusion, culminating in the final sample utilized for the TDT analysis (Figure 1). A significant sex difference (p<0.001) was noted among cleft types, with a higher prevalence of males in NSCL/P compared to NSCP, aligning with observations in other ethnic populations. This male predominance was consistent across all 3 ethnic groups, with males comprising 63.4% to 66.5% of the NSCL/P cases.

The baseline characteristics of these families are summarized in Table 1. Korean families exhibited a significantly older age distribution than both Asian and European families, with the mean age of affected children being 11.5 years (p<1×10^-5^). There was no significant sex difference among the affected Korean children. The average ages of Korean fathers and mothers were 44 years and 41 years, respectively. In contrast, the mean age of affected children in Asian families was 9.3 years, with fathers and mothers averaging 41.1 years and 38.1 years, respectively. European families had a mean age of 8.8 years for affected children, with fathers and mothers averaging 40.4 years and 37.1 years, respectively.

Two SNVs, rs7103685 and rs931953, deviated from HWE with p-values less than 0.005 in the Korean and Asian groups, respectively. Despite this, all SNVs satisfied stringent genotype quality control criteria, achieving high genotype call rates exceeding 99.8%. The missense mutation rs794066 exhibited the lowest MAF at 3.2%. The other SNVs were common variants, with MAFs ranging from 10.7% to 49.5%. These variants showed similar allele frequency trends across the 3 ethnic groups, though there were minor variations. For instance, rs4409845 had a MAF of 21.8% in the Korean group, compared to 12.7% in the Asian group and 10.7% in the European group (Table 2). Notably, SNVs rs3935406 and rs10892434 demonstrated strong LD (*r*^2^ > 0.8) in the parental data. MAF values for the Asian and European families were determined using the same minor allele designation as in the Korean population, facilitating direct comparisons across the 3 groups.

### Transmission disequilibrium test

The allelic TDT revealed a significant transmission distortion of the common C allele at the non-synonymous SNV rs7940667 (Gly361Val), with a p-value of 0.007. Haplotypes containing 2 to 4 SNVs, including rs7940667, did not enhance the statistically significant linkage in the presence of LD (0.10< p<0.05, data not shown), indicating that rs7940667 acts independently of other SNVs. The over-transmission of the C allele, compared to the alternative minor allele A (OR_C_ = 10.00 vs. OR_A_ = 0.10), remained statistically significant even after adjusting for multiple comparisons using the SNPSpD tool, with a threshold of p<0.0073. This consistent pattern of the major C allele being over-transmitted to affected children was observed in both Asian (OR_C_ =7.80, p=6.5×10^-7^) and European (OR_C_ =7.22, p=1.6×10^-10^) families (Table 3).

Genotype-based TDT analyses indicated that individuals with at least 1 C allele (A/C or C/C genotypes) are approximately 11 times more likely to develop NSCL/P than those with at least 1 A allele (OR_C/+_ = 11.11 vs. OR_A/+_ = 0.09, p=0.027) under a dominant model (Table 3). This association of the C/+ genotype was consistently observed in both Asian (OR_C/+_ = 8.33, p=1.410^-5^) and European (OR_C/+_ = 7.69, p=2.510^-8^) populations.

**Figure 2.**
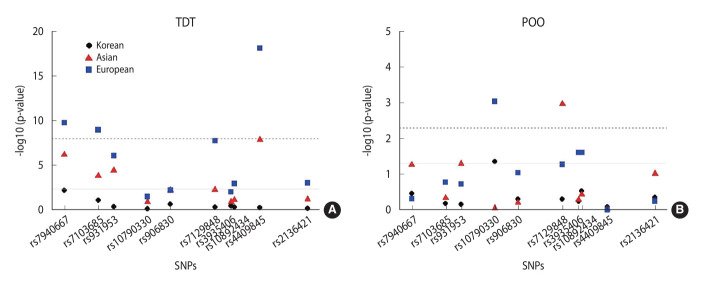
Evidence for the association of *PVRL1* with non-syndromic cleft lip with or without cleft palate in families of multiple ethnicities. (A) Transmission disequilibrium test (TDT) results. Each displayed p-value corresponds to the model (allelic or genotypic) that provided the best fit to the data for that single-nucleotide polymorphism (SNP). The red dashed line represents the genome-wide significance threshold (p<5×10^-8^), while the grey solid line indicates the Bonferroni-corrected significance threshold (p<0.01). (B) Parent-of-origin (POO) effect analysis. The red dashed line represents the Bonferroni-corrected significance threshold (p<0.01), and the grey solid line indicates the nominal significance threshold (p<0.05); p-values were derived from the POO likelihood ratio test.

Replication datasets, which included a larger number of families, identified significant transmission distortion in 7 additional SNVs associated with NSCL/P (rs7103685, rs931953, rs906830, rs7129848, rs10892434, rs4409845, and rs2136421), even after correcting for multiple comparisons (p<0.0073). Four SNVs (rs7940667, rs7103685, rs7129848, and rs4409845) demonstrated particularly strong associations (p<5×10^-8^) in at least 1 genetic model and ethnic group (Figure 2A). Notably, while the major A allele of rs4409845 showed no significant over-transmission to affected children in Korean families, it exhibited strong over-transmission in both Asian (p=1.4×10^-8^) and European affected families (p=7.3×10^-19^). The results from the genotypic TDT analysis of 10 *PVRL1* SNPs under additive, dominant, and recessive genetic models were compared across Korean, Asian, and European ethnic groups (Supplementary Material 1).

### Analysis of parent-of-origin effects

Our analysis of 10 *PVRL1* variants revealed significant maternal POO effects for several SNVs. Notably, the rs10790330-A allele showed a significant maternal POO effect in Korean families (Table 4). The OR for the allele transmitted maternally (OR_m_) was 1.5, indicating a significantly higher likelihood of the A allele being transmitted from the mother to the child compared to transmission from the father (OR_p_=0.77; p=0.044). This finding was replicated at the Bonferroni-corrected level of significance in European families (OR_m_ = 1.30 and OR_p_=0.91; p=9.0×10^-4^). Furthermore, the rs7129848-C allele exhibited a significant maternal POO effect in Asian families, achieving significance after Bonferroni correction (OR_m_ = 3.91 vs. OR_p_=1.42; p=0.001). However, this effect was only marginally significant in European families (OR_m_ = 2.65 vs. OR_p_=2.40; p=0.053) (Figure 2B). Additionally, 2 linked variants, rs3935406-A and rs10892434-C, showed nominal significance for maternal POO effects exclusively in European families (p=0.025).

## DISCUSSION

The initial study identified a rare homozygous non-sense mutation (W185X) in the *PVRL1* gene as the cause of rare autosomal recessive CLPED1 [6]. Subsequent analyses revealed that the W185X mutation, along with 2 other missense mutations (G361V and V395M), are more frequently observed in patients with NSCL/P. However, previous GWASs have not highlighted variants in the *PVRL1* gene as significant genetic factors for NSCL/P [11-13].

This study provides compelling evidence that *PVRL1* plays a role in the susceptibility to NSCL/P. Our TDT analysis showed a significant over-transmission of the C allele of the missense mutation, rs7940667 (G361V), to affected children in Korean families (p=0.007). This finding was strongly supported by subsequent TDT replication analyses in Asian (p=6.5×10^-7^) and European families (p=1.6×10^-10^). Additionally, carriers of the C allele exhibited a significantly increased risk of NSCL/P under a dominant model in all 3 ethnic groups, with ORs ranging from 7.7 (p=2.5×10^-8^) in European families to 11.1 (p=0.027) in Korean families.

Previous studies have reported conflicting findings. Over-transmission of the glycine allele was observed in 800 families from Iowa, Denmark, and the Philippines, including individuals with CP (p=0.005) [8]. In contrast, a Brazilian case-control study of NSCL/P did not show a significant difference [32]. It is crucial to compare allele frequencies across ethnic groups in TDT analysis to avoid confounding due to genetic differences between populations, validate findings, and ensure the generalizability of genetic associations. According to dbSNP, the rare A allele is more prevalent in African (YRI 27.5%) and European (7.1%) populations than in Asian populations (5.4%) (www.ncbi.nlm.nih.gov/snp). The analysis of Korean family data showed a lower frequency of the A allele in affected families (3.2%) compared to the parental dataset (5.0%), which is generally considered representative of the general population. This observation supports our finding that the major C allele of rs7940667 is over-transmitted to children affected by NSCL/P, suggesting it may be a risk allele for the disease.

Our initial analysis involving 142 NSCL/P families identified a significant association between rs7940667 and NSCL/P susceptibility. Subsequent investigations, which included larger cohorts of Asian and European families, provided additional evidence supporting the association of 8 SNVs with NSCL/P. These SNVs demonstrated strong TDT results, even after adjusting for multiple comparisons (p<0.0073). Notably, 4 of these SNVs—rs7940667, rs7103685, rs7129848, and rs4409845—showed particularly strong associations. Their p-values met the stringent threshold of p<5×10^-8^, a criterion typically used in genome-wide association studies, in at least 1 genetic model and ethnic group. This further reinforces the role of *PVRL1* in NSCL/P across diverse populations.

The *PVRL1* gene encodes nectin 1, a cell-cell adhesion molecule consisting of 517 amino acids. This molecule is essential for the formation of epithelial adherens junctions, which, in cooperation with cadherins, lead to the development of tight junctions in human epithelial cells [33]. *PVRL1* also functions as 1 of the 3 known primary receptors for alpha herpes virus binding and entry, known as herpes virus entry mediator C [34]. The expression of *PVRL1* in the developing face and palate indicates a possible connection between maternal viral infections during pregnancy and an increased risk of NSCL/P in offspring [8].

We further investigated the potential POO effects for *PVRL1* variants, which may indicate epigenetic modifications that influence differential gene expression. Significant maternal over-transmission of the rs10790330-A allele was observed in Korean families, a finding that was replicated in European families. Additionally, our analysis identified a significant maternal POO effect for the rs7129848-C allele in Asian families (p=0.001), aligning with the over-transmission observed in European families, albeit with marginal statistical significance (p=0.053).

The POO effect, influenced by imprinting mechanisms that differentially mark genes during gamete formation, remains poorly understood for most diseases. Imprinted genes play a critical role in the variation of complex traits [35]. Previous candidate gene studies have indicated that the parental origin affects the expression of certain genes involved in NSCL/P. For example, a study found a significant maternal over-transmission of *TGFA* single nucleotide polymorphisms (SNPs), such as rs3821261 (p=0.004) [21]. Conversely, another study observed paternal over-transmission of the rs12532 A allele of *MSX1* (p=0.025) [22]. However, previous genome-wide POO studies, examining over 2,000 caseparent trios, did not identify significant genome-wide POO effects for any SNPs. None of the SNPs located on chromosome 11, including those within *PVRL1*, reached a p<sub>POO<sub> significance threshold of 1×10^-5^ in the combined analysis of European and Asian NSCL/P trios [36,37]. This suggests that POO effects might be influenced by population-specific genetic backgrounds, gene-environment interactions such as maternal first-trimester viral infections, or maternal-fetal interactions, or they may represent subtle effects that require larger sample sizes to detect.

The present study, which includes a large dataset spanning multiple ethnic groups (884 families, 2,586 individuals), enhances the generalizability of our findings. These include the identification of significant SNVs and their transmission patterns. Notably, the non-synonymous SNV rs7940667 and the over-transmission of the C allele provide valuable insights into genetic risk factors.

Our study underscores the need for large-scale, ethnically targeted research to better understand the mechanisms behind POO effects and their relevance to NSCL/P. Mukhopadhyay et al. [13] also pointed out the challenges in replicating genetic associations across different populations, which can be attributed to variations in phenotype, sample size, or allele frequency. However, our study also has limitations that warrant further consideration. First, there was inconsistency in the family structures within our datasets. The Asian and European validation sets consisted solely of trios, whereas the Korean family groups included both 66 dyads and 76 trios. This variation, along with a smaller sample size relative to other ethnic groups, may have contributed to the lower statistical significance observed in the Korean family data. Second, our study did not consider factors such as phenotypic severity (e.g., laterality), parental environmental exposures, or the uterine environment, all of which are known to influence the NSCL/P phenotype. Lastly, there were differences between the Korean data and the validation data in terms of study design (targeted vs. genome-wide) and genotyping platform (Illumina Vera Code vs. Infinium HumanCore BeadChips). However, we believe these differences are unlikely to significantly affect the results.

Our findings contribute to understanding the genetic basis of NSCL/P. We provide robust evidence for the involvement of *PVRL1* in NSCL/P susceptibility, highlighting the importance of specific variants, including rs7940667, and demonstrating a potential role for POO effects. The finding of maternal over-transmission of particular *PVRL1* alleles underscores the complex relationship between genetic predisposition and environmental factors in the development of NSCL/P. This knowledge could guide future research efforts, including studies on gene-environment interactions and the potential impact of epigenetic modifications on *PVRL1* expression. With further validation, these insights could have significant implications for clinical practices, especially in the areas of disease risk prediction and genetic counseling.

## Figures and Tables

**Figure f3-epih-46-e2024069:**
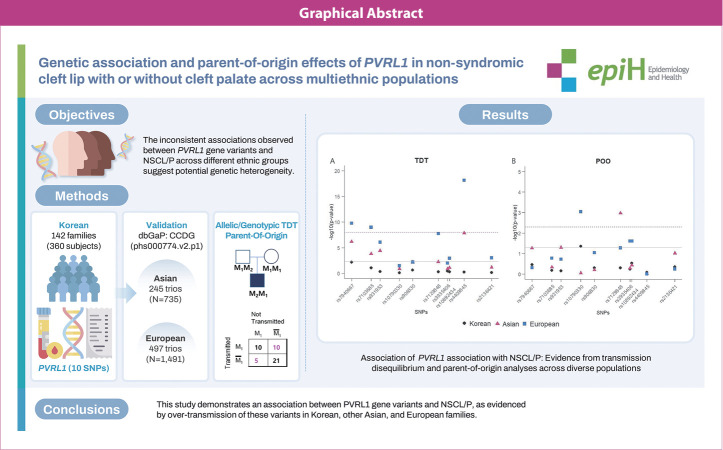


**Table 1.** Age and sex distribution of children with NSCL/P and their parents across 3 ethnic groups

Ethnic groups	Affected children		Parents		Subjects (trios), n
	n (%)	Age, mean±SD	n (%)	Age, mean±SD	
Korean	142 (100)	11.5±0.2	218 (100)	42.0±0.3	360 (142)
Male	90 (63.4)	11.4±0.3	94 (43.1)	43.6±0.4	
Female	52 (36.6)	11.6±0.4	124 (56.9)	40.8±0.4	
Asian	245 (100)	9.3±7.2	490 (100)	39.6±9.3	735 (245)
Male	163 (66.5)	8.6±6.4	245 (50.0)	41.1±9.4	
Female	82 (33.5)	10.6±8.5	245 (50.0)	38.1±9.1	
European	497 (100)	8.8±7.5	994 (100)	38.7±9.2	1,491 (497)
Male	319 (64.2)	8.9±7.2	497 (50.0)	40.4±9.0	
Female	178 (35.8)	8.5±8.1	497 (50.0)	37.1±9.1	

NSCL/P, non-syndromic cleft lip with or without cleft palate; SD, standard deviation.

**Table 2.** Characteristics of *PVRL1* gene variants in 3 ethnic groups^1^

SNP	M/m	Position (bp)^2^	Function	Korean (n=218)		Asian (n=490)		European (n=994)	
				MAF^3^	HWE (p)	MAF^3^	HWE (p)	MAF^3^	HWE (p)
rs7940667	C/A	119639934	Gly361Val	0.032	1.000	0.064	1.000	0.051	0.486
rs7103685	T/C	119652471	Intron	0.366	0.005	0.284	0.017	0.247	0.930
rs931953	A/G	119658195	Intron	0.477	0.118	0.297	0.003	0.242	0.629
rs10790330	A/G	119670622	Intron	0.500	1.000	0.475	0.224	0.508	0.723
rs906830	C/T	119679115	Intron	0.465	0.559	0.400	0.771	0.454	0.694
rs7129848	C/T	119695518	Intron	0.243	0.425	0.205	0.626	0.266	0.569
rs3935406	G/A	119701475	Intron	0.459	1.000	0.562	0.503	0.524	0.844
rs10892434	T/C	119702679	Intron	0.495	1.000	0.578	0.446	0.538	0.601
rs4409845	A/G	119712122	Intron	0.217	0.828	0.127	0.011	0.107	0.505
rs2136421	C/T	119729700	5’-975bp	0.449	0.549	0.525	0.162	0.554	0.430

SNP, single-nucleotide polymorphism; M/m, major/minor allele types in Korean parents; bp, base position; MAF, minor allele frequency; HWE, Hardy-Weinberg equilibrium p-value.

1 MAF and HWE (p) values were calculated using parental data only to represent the characteristics of each respective population.

2 Chromosomal position (bp) are shown based on the GRCh38 reference genome assembly.

3 For cross-group comparison, Asian and European parental MAF were derived using the Korean parental minor allele definition.

**Table 3.** Results of transmission disequilibrium test for 10 *PVRL1* SNPs in multiple-ethnicity family trios with NSCL/P

*PVRL1* 11q23.3	SNP	Allele R/NR	Allelic TDT		Genotypic TDT		
			T/NT^1^	p-value	Model^2^	OR (95% CI)	p-value
*Korean*	rs7940667	C/A	10:1	0.007	Dom	11.11 (1.32,100.00)	0.027
	rs7103685	T/C	47:36	0.275	Dom	1.61 (0.94, 2.78)	0.082
	rs931953	G/A	43:37	0.437	Add	1.19 (0.77, 1.84)	0.437
	rs10790330	A/G	38:36	0.816	Dom	1.09 (0.56, 2.13)	0.799
	rs906830	T/C	39:39	1.000	Rec	1.57 (0.75, 3.32)	0.233
	rs7129848	T/C	34:30	0.617	Rec	1.45 (0.51, 4.07)	0.486
	rs3935406	A/G	39:38	1.000	Dom	1.37 (0.69, 2.70)	0.375
	rs10892434	C/T	41:37	0.736	Rec	1.22 (0.66, 2.23)	0.530
	rs4409845	A/G	26:24	0.777	Add	1.20 (0.64, 2.27)	0.572
	rs2136421	C/T	41:37	0.736	Rec	1.15 (0.55, 2.44)	0.711
*Asian*	rs7940667	C/A	39:5	6.5×10^-7^	Dom	8.33 (3.13, 20.00)	1.4×10^-5^
	rs7103685	T/C	106:57	1.7×10^-4^	Add	1.85 (1.35, 2.56)	1.6×10^-4^
	rs931953	A/G	65:25	3.9×10^-5^	Add	2.63 (1.64, 4.17)	4.9×10^-5^
	rs10790330	G/A	66:60	0.656	Dom	1.52 (0.88, 2.63)	0.134
	rs906830	C/T	108:90	0.227	Rec	2.33 (1.27, 4.35)	0.006
	rs7129848	C/T	45:22	0.007	Add	2.04 (1.23, 3.45)	0.006
	rs3935406	A/G	102:93	0.567	Rec	1.47 (0.88, 2.44)	0.132
	rs10892434	C/T	102:98	0.832	Rec	1.59 (0.94, 2.70)	0.080
	rs4409845	A/G	75:19	1.4×10^-8^	Add	4.00 (2.38, 6.67)	8.9×10^-8^
	rs2136421	T/C	105:100	0.780	Rec	1.52 (0.97, 2.38)	0.068
*European*	rs7940667	C/A	65:9	1.6×10^-10^	Dom	7.69 (3.70, 14.30)	2.5×10^-8^
	rs7103685	T/C	225:112	1.1×10^-9^	Add	2.00 (1.61, 2.50)	1.6×10^-9^
	rs931953	A/G	147:73	8.6×10^-7^	Add	2.00 (1.52, 2.63)	1.0×10^-6^
	rs10790330	A/G	127:117	0.565	Dom	1.58 (1.04, 2.41)	0.034
	rs906830	C/T	246:193	0.013	Rec	1.59 (1.14, 2.22)	0.006
	rs7129848	C/T	124:49	1.8×10^-8^	Add	2.50 (1.82, 3.57)	3.7×10^-8^
	rs3935406	A/G	228:198	0.160	Rec	1.54 (1.11, 2.13)	0.010
	rs10892434	C/T	240:198	0.050	Rec	1.75 (1.25, 2.50)	0.001
	rs4409845	A/G	150:30	7.3×10^-19^	Add	5.00 (3.33, 7.14)	8.5×10^-16^
	rs2136421	T/C	221:212	0.701	Rec	1.82 (1.28, 2.63)	9.5×10^-4^

SNP, single-nucleotide polymorphism; NSCL/P, non-syndromic cleft lip with or without cleft palate; R/NR, risk/non-risk allele type; T/NT, numbers of transmitted/non-transmitted families; TDT, transmission disequilibrium test; OR, odds ratio; CI, confidence interval; Add, additive; Dom, dominant; Rec, recessive.

1 Allelic transmission distortion was assessed for each SNP by comparing the number of families with the minor allele transmitted to offspring versus those with the allele not transmitted; ORs were then calculated based on the T/NT allele counts for the over-transmitted allele.

2 Genotypic TDT was evaluated for the risk allele under 3 genetic models: Add, Dom, and Rec; The ORs (95% CIs) and p-values are shown for the model demonstrating the strongest association signal.

**Table 4.** Parent-of-origin effects for 10 SNPs in the *PVRL1* gene in multiple-ethnicity family trios with NSCL/P

*PVRL1* 11q23.3	SNP	Allele R/NR	Maternal TDT			Paternal TDT			PO-LRT^2^ p-value
			T/NT^1^	OR_m_	p-value	T/NT^1^	OR_p_	p-value	
Korean	rs7940667	C/A	6:1	6.00	0.102	4:0	NA	0.083	0.350
	rs7103685	T/C	25.5:18.5	1.38	0.144	21.5:17.5	1.23	0.491	0.667
	rs931953	G/A	21:19	1.11	1.000	22:18	1.22	0.467	0.703
	rs10790330	A/G	21:14	1.50	0.059	17:22	0.77	0.394	0.044
	rs906830	T/C	17:19	0.89	0.617	22:20	1.10	0.670	0.502
	rs7129848	T/C	16:16	1.00	0.819	18:14	1.29	0.251	0.495
	rs3935406	A/G	19.5:16.5	1.18	0.819	19.5:21.5	0.91	0.532	0.574
	rs10892434	C/T	21:15	1.40	0.491	20:22	0.91	0.414	0.295
	rs4409845	A/G	11:11	1.00	0.796	15:13	1.15	0.513	0.813
	rs2136421	C/T	23:18	1.28	0.394	18:19	0.95	0.808	0.450
Asian	rs7940667	C/A	24:5	4.80	0.002	15:0	NA	5.3×10^-4^	0.054
	rs7103685	T/C	54.5:30.5	1.79	0.199	51.5:26.5	1.94	0.123	0.461
	rs931953	A/G	31.5:12.5	2.52	0.011	33.5:12.5	2.68	0.005	0.050
	rs10790330	G/A	30.5:28.5	1.07	0.683	35.5:31.5	1.13	0.480	0.890
	rs906830	C/T	54:42	1.29	1.000	54:48	1.13	0.376	0.632
	rs7129848	C/T	21.5:5.5	3.91	0.005	23.5:16.5	1.42	0.590	0.001
	rs3935406	A/G	49.5:41.5	1.19	1.000	52.5:51.5	1.02	0.317	0.516
	rs10892434	C/T	49.5:43.5	1.14	0.752	52.5:54.5	0.96	0.174	0.369
	rs4409845	A/G	39:11	3.55	0.001	36:8	4.50	4.1×10^-4^	0.955
	rs2136421	T/C	55.5:51.5	1.08	0.686	49.5:48.5	1.02	0.376	0.096
European	rs7940667	C/A	33:4	8.25	1.4×10^-5^	32:5	6.40	6.2×10^-5^	0.486
	rs7103685	T/C	112:62	1.81	0.034	113:50	2.26	3.8×10^-4^	0.170
	rs931953	A/G	76:41	1.85	0.056	71:32	2.22	0.008	0.189
	rs10790330	A/G	69.5:53.5	1.30	0.027	57.5:63.5	0.91	0.396	9.0×10^-4^
	rs906830	C/T	133.5:94.5	1.41	0.012	112.5:98.5	1.14	1.000	0.092
	rs7129848	C/T	67.5:25.5	2.65	0.002	56.5:23.5	2.40	0.025	0.053
	rs3935406	A/G	127.5:96.5	1.32	0.009	100.5:101.5	0.99	0.497	0.025
	rs10892434	C/T	135.5:94.5	1.43	0.010	104.5:103.5	1.01	0.118	0.025
	rs4409845	A/G	79:15	5.27	2.4×10^-8^	71:15	4.73	8.5×10^-7^	0.991
	rs2136421	T/C	115.5:102.5	1.13	0.361	105.5:109.5	0.96	0.409	0.565

SNP, single-nucleotide polymorphism; M/m, major/minor allele type; PO-LRT, parent-of-origin likelihood ratio test; ORm/ORp, odds ratio of maternally/paternally transmitted allele; T/NT, numbers of transmitted/not-transmitted allele count; TDT, transmission disequilibrium test.

1 Allelic transmission distortion was assessed for each SNP, comparing transmission rates for maternal and paternal alleles by comparing; ORs were then calculated based on the T/NT allele counts for the over-transmitted allele.

2 POO effects were evaluated using PO-LRT, comparing the transmission rates of maternal and paternal alleles.

## Supplementary materials

Supplementary Material 1. Results of genotypic transmission disequilibrium test for ten *PVRL1* SNPs in multi-ethnic family trios with NSCL/P
