# Supplementary material for: Genetic associations and parent-of-origin effects of PVRL1 in non-syndromic cleft lip with or without cleft palate across multiple ethnic populations
Source: Epidemiol Health. 2024 Aug 9;46:e2024069. doi: 10.4178/epih.e2024069 (PMC11576525; doi:10.4178/epih.e2024069)
Supplement: Supplementary Material 1. — Results of genotypic transmission disequilibrium test for ten PVRL1 SNPs in multi-ethnic family trios with NSCL/P [file epih-46-e2024069-Supplementary-1.docx]

| **Supplementary Material 1.** Results of genotypic transmission disequilibrium test for ten *PVRL1* SNPs in multi-ethnic family trios with NSCL/P | | | | | | | | |
| --- | --- | --- | --- | --- | --- | --- | --- | --- |
| *PVRL1* | SNP | Allele | Genotypic TDT^2^ | | | | | |
| 11q23.3 |  | R/NR | Additive | | Dominant | | Recessive | |
|  |  |  | OR | P | OR | *P* | OR | *P* |
| *Korean* | rs7940667 | C/A | 10.0 | 0.028 | 11.1 | 0.027 | NA | NA |
|  | rs7103685 | T/C | 1.31 | 0.276 | 1.61 | 0.082 | 0.81 | 0.601 |
|  | rs931953 | G/A | 1.19 | 0.437 | 1.18 | 0.622 | 1.27 | 0.480 |
|  | rs10790330 | A/G | 1.06 | 0.816 | 1.09 | 0.799 | 1.03 | 0.928 |
|  | rs906830 | T/C | 1.00 | 1.000 | 0.76 | 0.361 | 1.57 | 0.233 |
|  | rs7129848 | T/C | 1.13 | 0.617 | 1.07 | 0.823 | 1.45 | 0.486 |
|  | rs3935406 | A/G | 1.03 | 1.000 | 0.73 | 0.375 | 1.30 | 0.415 |
|  | rs10892434 | C/T | 1.11 | 0.736 | 0.93 | 0.854 | 1.22 | 0.530 |
|  | rs4409845 | A/G | 1.08 | 0.777 | 1.20 | 0.572 | 0.71 | 0.603 |
|  | rs2136421 | C/T | 1.11 | 0.736 | 1.05 | 0.874 | 1.15 | 0.711 |
| *Asian* | rs7940667 | C/A | 7.80 | 1.5×10^-5^ | 8.33 | 1.4×10^-5^ | NA | NA |
|  | rs7103685 | T/C | 1.85 | 1.6×10^-4^ | 1.75 | 0.003 | 5.26 | 0.007 |
|  | rs931953 | A/G | 2.60 | 4.9×10^-5^ | 2.50 | 3.9×10^-4^ | 9.09 | 0.034 |
|  | rs10790330 | G/A | 1.10 | 0.593 | 1.52 | 0.134 | 0.80 | 0.439 |
|  | rs906830 | C/T | 1.20 | 0.201 | 0.93 | 0.700 | 2.33 | 0.006 |
|  | rs7129848 | C/T | 2.05 | 0.006 | 2.00 | 0.013 | 4.35 | 0.161 |
|  | rs3935406 | A/G | 1.10 | 0.519 | 0.93 | 0.725 | 1.47 | 0.132 |
|  | rs10892434 | C/T | 1.04 | 0.777 | 0.82 | 0.308 | 1.59 | 0.080 |
|  | rs4409845 | A/G | 3.95 | 8.9×10^-8^ | 4.00 | 2.3×10^-7^ | NA | NA |
|  | rs2136421 | T/C | 1.05 | 0.727 | 0.76 | 0.215 | 1.52 | 0.068 |
| *European* | rs7940667 | C/A | 7.22 | 2.7×10^-8^ | 7.69 | 2.5×10^-8^ | NA | NA |
|  | rs7103685 | T/C | 2.01 | 1.6×10^-9^ | 2.08 | 1.6×10^-8^ | 2.70 | 0.004 |
|  | rs931953 | A/G | 2.01 | 1.0×10^-6^ | 2.08 | 4.7×10^-6^ | 2.86 | 0.018 |
|  | rs10790330 | A/G | 1.09 | 0.522 | 1.58 | 0.034 | 0.80 | 0.268 |
|  | rs906830 | C/T | 1.27 | 0.012 | 1.18 | 0.226 | 1.59 | 0.006 |
|  | rs7129848 | C/T | 2.53 | 3.7×10^-8^ | 2.33 | 9.6×10^-6^ | 20.00 | 0.003 |
|  | rs3935406 | A/G | 1.15 | 0.146 | 0.97 | 0.832 | 1.54 | 0.010 |
|  | rs10892434 | C/T | 1.21 | 0.045 | 1.00 | 1.000 | 1.75 | 0.001 |
|  | rs4409845 | A/G | 5.00 | 8.5×10^-16^ | 5.26 | 1.3×10^-15^ | NA | NA |
|  | rs2136421 | T/C | 1.04 | 0.665 | 0.74 | 0.029 | 1.82 | 9.5×10^-4^ |

NA, not available; R/NR, risk/non-risk allele type; OR, odds ratio; SNP, single nucleotide polymorphism; T/NT, numbers of transmitted/non-transmitted families; TDT, transmission disequilibrium test.

^1^ Allelic transmission distortion was assessed for each SNP by comparing the number of families with the minor allele transmitted to offspring versus those with the allele not transmitted. Odds ratios (ORs) were then calculated based on the transmission/non-transmission (T/NT) allele counts for the over-transmitted allele.

^2^ Genotypic TDT was evaluated for the risk allele under three genetic models: additive, dominant, and recessive.
